# Rapid differentiation of hiPSCs into functional oligodendrocytes using an *OLIG2* synthetic modified messenger RNA

**DOI:** 10.1038/s42003-022-04043-y

**Published:** 2022-10-14

**Authors:** Jian Xu, Zhihua Yang, Rui Wang, Fumei He, Rong Yan, Yidi Zhang, Liying Yu, Wenbin Deng, Yichu Nie

**Affiliations:** 1 School of Pharmaceutical Sciences (Shenzhen), Shenzhen Campus of Sun Yat-sen University, Shenzhen, 518107 China; 2 Stroke Center, The Fifth Affiliated Hospital of Guangzhou Medical University, Guangzhou, 510799 China; 3 Clinical Research Institute, The First People’s Hospital of Foshan, Foshan, 528000 China

**Keywords:** Induced pluripotent stem cells, Demyelinating diseases

## Abstract

Transcription factors (TFs) have been introduced to drive the highly efficient differentiation of human-induced pluripotent stem cells (hiPSCs) into lineage-specific oligodendrocytes (OLs). However, effective strategies currently rely mainly on genome-integrating viruses. Here we show that a synthetic modified messenger RNA (smRNA)-based reprogramming method that leads to the generation of transgene-free OLs has been developed. An smRNA encoding a modified form of *OLIG2*, in which the serine 147 phosphorylation site is replaced with alanine, *OLIG2*^S147A^, is designed to reprogram hiPSCs into OLs. We demonstrate that repeated administration of the smRNA encoding *OLIG2* ^S147A^ lead to higher and more stable protein expression. Using the single-mutant *OLIG2* smRNA morphogen, we establish a 6-day smRNA transfection protocol, and glial induction lead to rapid NG2^+^ OL progenitor cell (OPC) generation (>70% purity) from hiPSC. The smRNA-induced NG2^+^ OPCs can mature into functional OLs in vitro and promote remyelination in vivo. Taken together, we present a safe and efficient smRNA-driven strategy for hiPSC differentiation into OLs, which may be utilized for therapeutic OPC/OL transplantation in patients with neurodegenerative disease.

## Abstract (web summary)

The use of synthetic modified messenger RNA (smRNA) allows for the differentiation of human-induced pluripotent stem cells (hiPSCs) into lineage-specific oligodendrocytes.

## Introduction

Oligodendrocytes (OLs), which are myelinating cells of the central nervous system (CNS), are extremely promising therapeutic targets for cell replacement-based therapies for myelin loss or dysfunction, such as in patients with multiple sclerosis and white matter ischemic injury^1–4^. Reprogramming human-induced pluripotent stem cells (hiPSCs) to OLs is a profoundly promising approach for disease modeling, drug development, and OL transplantation based therapeutic approaches. Research advances have made it possible to generate surface antigen O4 (O4)-positive and myelin basic protein (MBP)-positive OLs from hiPSCs within only ~20 days by overexpressing key transcription factors (TFs)^5^. However, the traditional approach to achieving TF overexpression generally involves ectopic virus-mediated gene delivery, and viral integration into the genome initially is an obstacle to the therapeutic use of OLs. Therefore, synthetic modified messenger RNAs (smRNAs) were developed in vitro to diminish the innate immune response and improve the delivery of genetic material that can be efficiently translated into specific functional proteins into mammalian cells^6–8^. In contrast to DNA-based gene manipulation, the introduction of smRNA carries no risk of genomic integration, as smRNAs are translated in the cytoplasm without being delivered into the nucleus, indicating that smRNA delivery is a safer and more efficient method for inducing protein expression.

Instability and a small window for inducing protein expression are the major obstacles when using smRNAs for cellular reprogramming. For mRNAs to be effectively translated in vitro, the 5’-terminal m7GpppG cap and the 3’-terminal poly(A) sequence need to be incorporated into the mRNAs structure for in vitro transcription (IVT). 5-Methyl-cTP, pseudo/ψ-UTP and other modified nucleotides have also been incorporated into mRNA to reduce immunogenicity and increase stability^9^. An smRNA was used to directly reprogram the fate of human somatic cells into hiPSCs^10^. Warren et al. employed smRNAs to drive the expression of “reprogramming” factors and successfully developed a method for hiPSC generation^11^. In particular, this method of inducing protein expression mediated by smRNAs has the potential to become a very useful technology for cell-based therapies and regenerative medicine. Moreover, smRNAs have been used to direct the fate of reprogrammed hiPSCs into tissue-specific cell types^12,13^; however, smRNA-driven differentiation hiPSCs into OLs has been largely unreported. We and other researchers have sought to develop protocols to achieve uniform and reproducible cultures of hiPSC-derived OL progenitor cells (OPCs)^5,14–18^. We identified factors that facilitate the differentiation of many OPCs derived from hiPSCs, optimized methods, defined conditions, and tested the survival and differentiation of the cells in animal models. Transplantation of iPSC-derived OPCs that have been better “instructed” to follow the OL lineage may facilitate the recovery of patients with CNS diseases^14,19^. Regulating the expression of the TF *OLIG2* affects a major regulatory transcription pathway in OL genesis^20,21^. Overexpression of *OLIG2* in primary neural stem cells (NSCs) has been reported to facilitate myelinating OL generation and contribute to remyelination of the corpus callosum in mice with experimental allergic encephalomyelitis (EAE)^22^. Although previous studies have shown that the expression of oligodendroglial lineage marker genes, such as nerve/glial antigen 2 (NG2, also known as CSPG4) and platelet-derived growth factor receptor alpha (PDGFRa, also known as CD140a), was not induced following overexpression of the TF *OLIG2* alone in neural progenitor cells (NPCs)^17^, our published data have shown that GANT61, a small Shh inhibitor, can abolish the development of motor neurons (MNs) and improve OL specification by regulating the posttranslational phosphorylation of the *OLIG2* protein^23^. Therefore, it seems that the significance of *OLIG2* overexpression in OL specification should be further emphasized. Indeed, posttranslational modifications (PTM) of the *OLIG2* protein has been shown to influence cell fate transitions. Li et al. reported that a specific serine residue (serine 147) of *OLIG2* is essential for MN-OL switching. Replacing serine 147 with an alanine residue (S147A) was found to abolish MN production without preventing OL formation^24^. We expect that we will establish better protocols for inducing hiPSC differentiation into OLs through the forced expression of an smRNA coding mutant *OLIG2* (*OLIG2*^S147A^) and that this *OLIG2* smRNA may be used to develop more efficient protocols for inducing mRNA-driven differentiation of hiPSCs into OLs.

In this study, we established a novel approach for inducing efficient and rapid hiPSC differentiation into OL lineage-specific cells through repeated administration of an smRNA and found that phosphosite modification of OLIG2 effectively promoted the efficiency of this smRNA-driven hiPSC differentiation process. This novel single virus-free smRNA-driven method will broaden the application of OL replacement therapy in various diseases involving myelin injury.

## Results

### An smRNA encoding *OLIG2* with a phosphorylation site modification drives highly efficient oligodendroglial lineage cell differentiation of hiPSC-derived NPCs

**Fig. 1:**
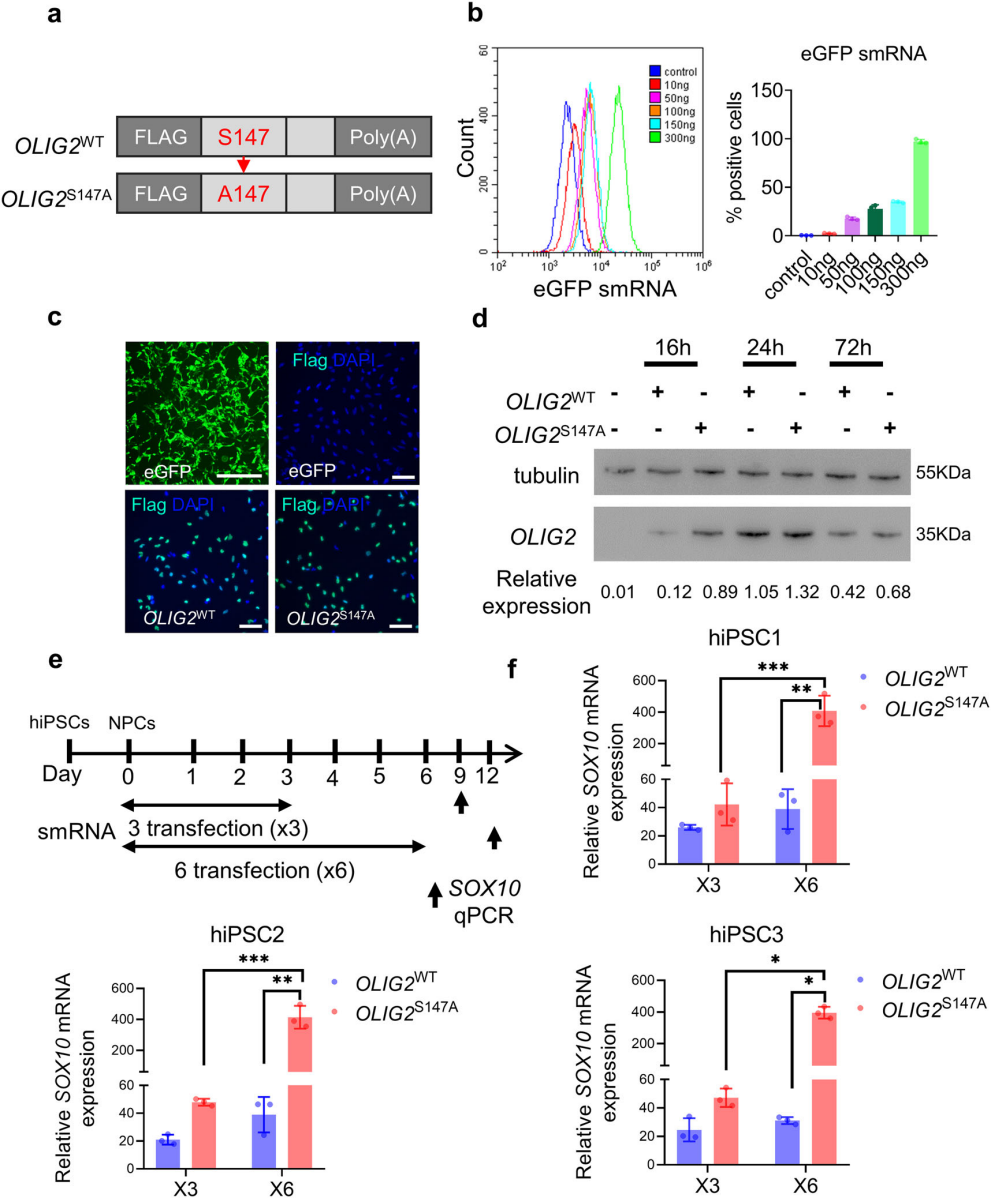
An smRNA coding *OLIG2* with phosphosite modification enhances protein expression and can induce differentiation into oligodendroglial lineage cells. **a** Diagram of the smRNAs encoding wild-type (*OLIG2*^WT^) and phosphosite-modified *OLIG2* (*OLIG2*^S147A^). **b** Analysis of the smRNA transfection efficiency in hiPSC-derived NPCs transfected with 10, 50, 100, 150, and 300 ng of eGFP smRNA at 24 h posttransfection. A representative overlay histogram is presented. The transfection efficiency of NPCs treated with 300 ng of eGFP smRNA was greater than 95% (*n* = 3). **c** hiPSC-derived NPCs were transfected with eGFP, *OLIG2*^WT^ or *OLIG2*^S147A^ (3XFlag-tagged) smRNA as indicated. Twenty-four hours after transfection, fluorescence microscopy showed a large number of eGFP^+^ or Flag^+^ cells (DAPI: nuclei; scale bar, 200 μm). **d** hiPSC1-derived NPCs were transfected with smRNA, and total proteins were harvested at the indicated time points for western blotting for OLIG2. The fold change in protein expression normalized to tubulin expression is shown below each lane. The representative quantitative data are from three independent experiments. **e** hiPSC-derived NPCs underwent three (×3) or six (×6) daily smRNA transfections. Total RNA was isolated for qPCR analysis of *SOX10* expression on day 9 and day 12. **f** qPCR analysis of *SOX10* mRNA expression in three different hiPSC lines at 3 days after smRNA transfection. *OLIG2*^S147A^ smRNA more potently induced the expression of the downstream target gene of *OLIG2* (*SOX10*) than wild-type smRNA (*n* = 3; *p < 0.05, **p < 0.01 and ***p < 0.001 using two-tailed Student’s *t* test).

We delivered smRNA to induce the expression of a mutant *OLIG2* protein that recapitulates the induction of oligodendroglial TFs during OL development. Two vectors carrying mRNAs encoding wild-type *OLIG2* and mutant *OLIG2* with a specific serine-to-alanine modification were synthesized for T7 promoter-driven IVT (Figs. 1a and S1). Before testing these *OLIG2* smRNAs, we first determined the efficiency of smRNA delivery into NPCs derived from hiPSCs. Using a cationic liposome-based delivery system, we introduced eGFP smRNA into NPCs as an in vitro tracer. As shown in Fig. 1b, transfection of 300 ng of eGFP smRNA along into NPCs resulted in a transfection efficiency greater than 95%. Moreover, immunofluorescence staining for Flag revealed Flag-tagged *OLIG2*^WT^ and Flag-tagged *OLIG2*^S147A^ expression in NPCs at 24 h posttransfection (Fig. 1c). Based on these results, these smRNAs were successfully delivered into NPCs and translated into proteins. Next, smRNA-induced cytotoxicity, which would decrease the efficiency of cellular reprogramming, was determined using the CCK-8 assay. No significant cytotoxicity was observed in smRNA-transfected cells compared to untransfected cells (Fig. S2). These results supported our strategy of utilizing mutant *OLIG2* smRNA to drive hiPSC-derived NPC differentiation into OLs.

Blocking *OLIG2* phosphorylation at serine 147 has been shown to abolish MN production without preventing OL production and to trigger the switch from an MN fate to an OL fate^24^. Using our optimized strategy for smRNA synthesis and transfection, we used smRNAs encoding wild-type and phosphorylation site-modified *OLIG2* to determine which protein induces greater protein expression and OL specification. The smRNA encoding *OLIG2* with the S147A phosphorylation site modification resulted in 1.2-fold higher protein expression than the smRNA encoding wild-type *OLIG2* at 16 h posttransfection, and this difference peaked 24 h after smRNA transfection (Fig. 1d). We then asked whether *OLIG2*^WT^ smRNA or *OLIG2*^S147A^ smRNA more strongly induced the expression of validated downstream targets of *OLIG2*, such as *SOX10*, which is required for OL differentiation and maturation^25^. As shown in Fig. 1e, cells underwent three or six daily transfections of smRNAs to determine the most suitable and efficient differentiation strategy. NPCs derived from the three different hiPSC lines were plated in 12-well plates precoated with the reduced growth factor Matrigel/laminin, and *SOX10* mRNA expression was measured. The qPCR results suggested that six daily transfections of *OLIG2*^S147A^ induced 9.8-fold higher *SOX10* mRNA expression than three daily transfections in hiPSC-derived NPCs. Similarly, we found that *OLIG2*^S147A^ smRNA drove higher expression of a OL lineage marker gene (*SOX10*) than *OLIG2*^WT^ smRNA in hiPSC2- and hiPSC3-derived NPCs (Fig. 1f). Type I interferon (IFN) is considered a marker for antiviral defense responses mediated by interferon-γ- and NF-kB-dependent pathways in mammalian cells^26^. The expression of the signaling marker IFN was transient, as evidenced by significant downregulation of IFN 48 h after six daily smRNA transfections (Fig. S3). Thus, the smRNA encoding *OLIG2* with a phosphorylation site modification was a better driver of OL differentiation from hiPSC-derived NPCs than its respective wild-type counterpart.

### smRNA-induced hiPSC differentiation into OPCs

**Fig. 2:**
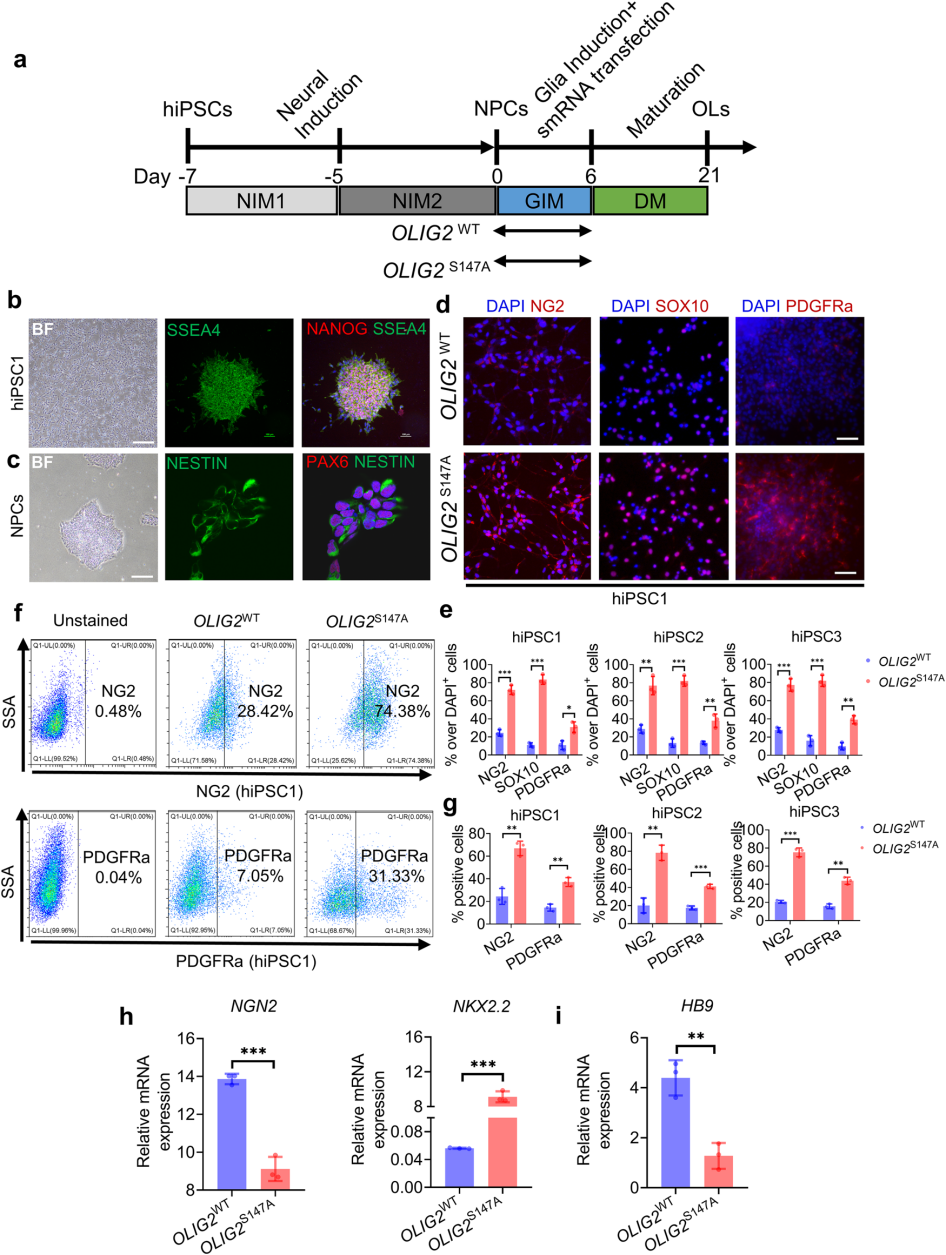
A highly efficient strategy for generating smRNA-induced oligodendroglial lineage cells from hiPSC-derived NPCs. **a** Diagram of the six daily smRNA differentiation protocols for hiPSCs. **b** Representative images of immunofluorescence staining for the human NANOG and SSEA4 proteins in cultured hiPSC1 cells (scale bar, 200 μm). **c** Representative images of immunofluorescence staining for the human PAX6 and NESTIN proteins in hiPSC1-derived NPCs (scale bar, 20 μm). **d** Representative images of immunofluorescence staining for NG2, SOX10, and PDGFRα in smRNA-transfected cells of the hiPSC1 line on day 6 (scale bar, 50 μm). **e** Quantification of the percentage of NG2, SOX10 and PDGFRα on day 6 after smRNA transfection (*n* = 3; *p < 0.05, **p < 0.01, ***p < 0.001 using two-tailed Student’s *t* test). *OLIG2*^S147A^ mRNA induced greater NG2^+^/SOX10^+^/PDGFRα^+^ OPC production than *OLIG2*^WT^ smRNA. **f** Representative flow cytometry analyses of the expression of NG2 and PDGFRα in smRNA-transfected cells of the hiPSC1 line on day 6. **g** Quantification of NG2^+^ and PDGFRα^+^ cells among *OLIG2*^WT^ and *OLIG2*^S147A^ smRNA-transfected cells of the three different hiPSC lines on day 6 (*n* = 3, **p < 0.01, ***p < 0.001 using two-tailed Student’s *t* test). **h** The mRNA expression levels of a proneural marker gene (*NGN2*) and oligodendroglial lineage marker gene (*NKX2.2*) involved in OL differentiation on day 6 after smRNA transfection (*n* = 3; ***p < 0.001 using two-tailed Student’s *t* test). **i** The mRNA expression levels of the MN-specific marker gene *HB9* on day 6 after smRNA transfection (*n* = 3; **p < 0.01 using two-tailed Student’s *t* test).

Since six daily smRNA transfections resulted in higher OL differentiation potential, we next established 6-day smRNA transfection schemes for mRNA-induced differentiation of hiPSCs into OLs (Fig. 2a). First, *NANOG-* and *SSEA4*-positive cells of the hiPSC1 line (Fig. 2b) were differentiated into NPCs by culture in NIM for 7 days, and the cultured cells expressed *PAX6* and *NESTIN* (Fig. 2c). One day after smRNA transfection, the culture medium was replaced with GIM containing different morphogens (T3, SAG and PDGFR-AA). After 6 days, the GIM was changed to DM lacking PDGF-AA and SAG to promote OL generation and maturation. Six days after smRNA treatment and GIM transfection, smRNA-induced NG2^+^ OPCs were identified. In contrast, NPCs transfected with or without eGFP smRNA failed to attach or survive in culture with GIM; however, NPCs transfected with *OLIG2*^S147A^ smRNA promoted NG2^+^/SOX10^+^/PDGFRa^+^ OPC generation to a greater degree than NPCs transfected with *OLIG2*^WT^ smRNA (Fig. 2d, e). To verify the reproducibility of this phenomenon in a variety of hiPSC lines, the two other hiPSC lines also underwent smRNA-mediated OL differentiation. Similarly, there were more NG2-, SOX10-, and PDGFRα-positive cells among hiPSC2- or hiPSC3-derived NPCs transfected with *OLIG2*^S147A^ smRNA than those transfected with OLIG2^WT^ smRNA (Fig. 2e). Additionally, cell death was not increased in hiPSC-derived NPCs transfected with *OLIG2*^WT^ or *OLIG2*^S147A^ smRNA compared with eGFP smRNA-transfected and untransfected cells after 6 daily transfections (Fig. S4). We analyzed the cultured cells after 6 daily transfections using flow cytometry to further characterize OPC generation induced by *OLIG2*^S147A^ smRNA. The *OLIG2*^S147A^ smRNA-treated cells were largely NG2^+^ cells (74.94 ± 2.68%), unlike the *OLIG2*^WT^ smRNA-transfected cells (27.72 ± 1.25%, Fig. 2f, g). PDGFRα, another marker, was utilized for OPC identification^27^. A total of 32.82% ± 2.68% of *OLIG2*^S147A^ smRNA-treated cells were PDGFRa^+^ OPCs; however, only 6.68 ± 1.25% of *OLIG2*^WT^ smRNA-treated cells were PDGFRα^+^ OPCs (Fig. 2f, g). These results were consistent with those for the other two hiPSC lines showing that OLIG2^S147A^ smRNA induced hiPSC differentiation into OPCs. Blocking OLIG2 phosphorylation downregulates the expression of the MN-specific marker *NGN2*, which results in the initiation of OL production^24^. We thus determined *NGN2* mRNA expression levels in *OLIG2*^WT^ smRNA and *OLIG2*^S147A^ smRNA-treated cultures and found that *OLIG2*^S147A^ smRNA downregulated *NGN2* mRNA expression to a significantly greater extent than *OLIG2*^WT^ smRNA but upregulated the mRNA expression of *NKX2.2*, which is required for OPC/OL differentiation (Fig. 2h). qPCR further showed that the mRNA expression level of *HB9*, a TF downstream of *NGN2*, was also decreased in *OLIG2*^S147A^ smRNA-induced OPCs (Fig. 2i). Taken together, these results suggested that the smRNA encoding *OLIG2* with the phosphorylation site modification facilitated highly efficient OPC generation.

### Maturation of *OLIG2* smRNA-induced OPCs into functional OLs

**Fig. 3:**
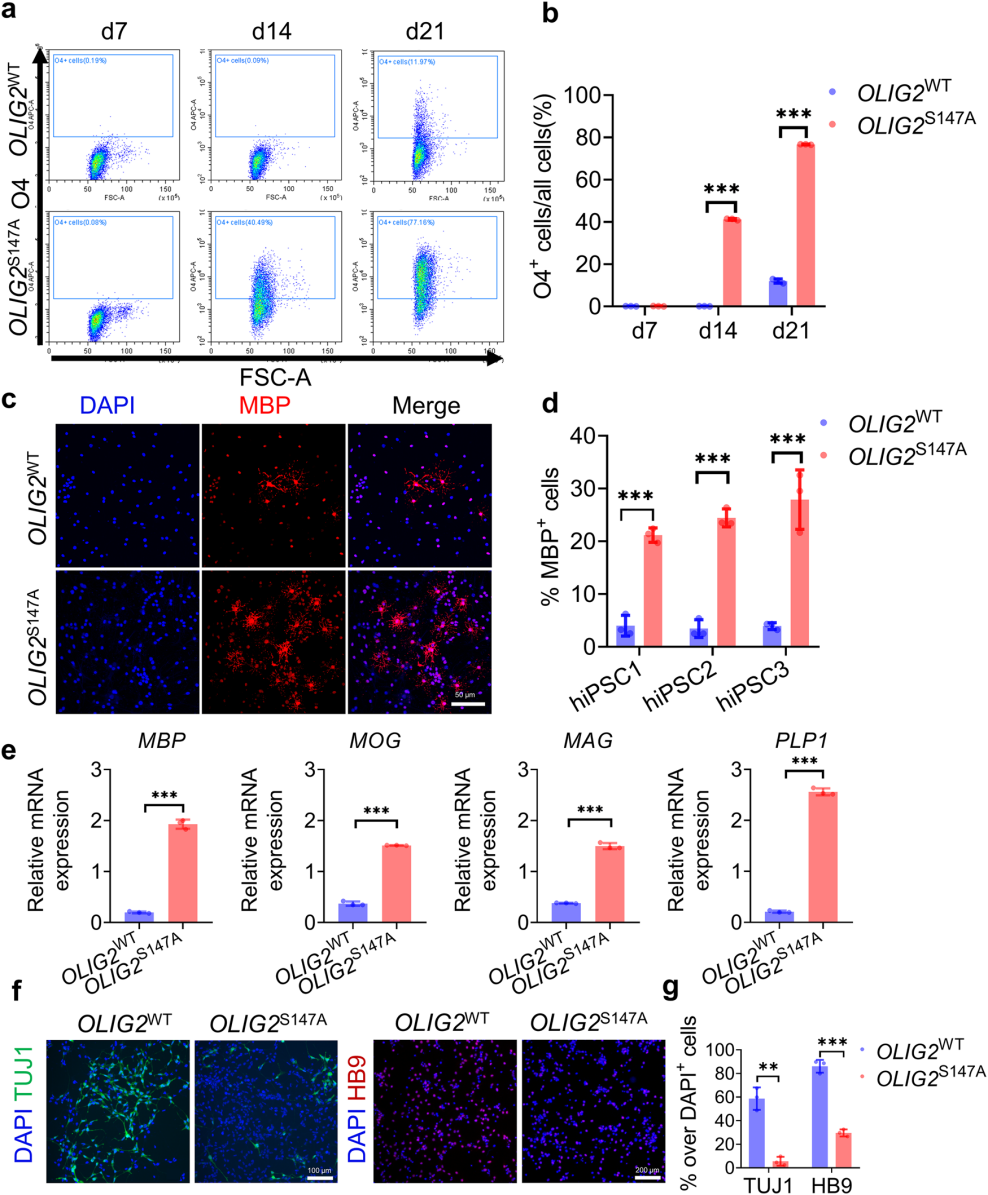
Maturation of *OLIG2* smRNA-treated OPCs into myelinating OLs. **a** Representative flow cytometry analyses of the expression of O4 in the *OLIG2*^WT^ and *OLIG2*^S147A^ smRNA-transfected cells at 7, 14 and 21 days after transfection. **b** Quantification of O4^+^ cells among *OLIG2*^WT^ and *OLIG2*^S147A^ smRNA-transfected cells at 7, 14 and 21 days after transfection (*n* = 3, ***p < 0.001 using two-tailed Student’s *t* test). **c** Representative images of immunostaining for MBP in cultured cells of the three different hiPSC lines on day 21 after smRNA transfection (scale bar, 50 μm). **d** Quantification of the number of MBP-positive cells among cells of the hiPSC1 line on day 21 after smRNA transfection (*n* = 3; ***p < 0.001 using two-tailed Student’s *t* test). **e** mRNA expression levels on day 21 of the OL differentiation protocol (*n* = 3, ***p < 0.001 using two-tailed Student’s *t* test). **f** Representative images of immunostaining for TUJ1 and HB9 in hiPSC1-derived cells on day 21 after smRNA transfection (scale bar, 100 μm (left) and 200 μm (right)). **g** Quantification of TUJ1- and HB9-positive cells among hiPSC1-derived cells (*n* = 3, **p < 0.01, ***p < 0.001 using two-tailed Student’s *t* test).

Differentiation of OPCs into mature OLs upon stimulation with both extrinsic and intrinsic factors is a key event required for axon myelination in the CNS. We next investigated whether smRNA-induced OPCs subsequently matured into functional OLs. The O4 epitope, which is a mature OPC or pre-OL marker, was utilized to assess the terminal differentiation of OPCs^28^. To determine the kinetics, efficiency, and yield of mRNA-mediated OL lineage specification, we performed weekly flow cytometry assays to evaluate O4^+^ OPC/OL generation in our culture system. As shown in Fig. 3a, b, the percentage of *OLIG2*^S147A^ smRNA-induced O4^+^ cells was 40.49 ± 2.54% on day 14 and increased to 77.16 ± 6.24% by day 21; however, only 0.09 ± 1.25% O4^+^ cells were observed on day 7, and 11.97 ± 2.24% O4^+^ cells were observed on day 21 in the *OLIG2*^WT^ smRNA-treated cultures. The expression of *MBP*, a membrane actin-binding protein in CNS myelin, is a critical marker of OLs and multilayered compact myelin^29^. Immunofluorescence staining for MBP confirmed that unlike *OLIG2*^WT^ smRNA transfection, *OLIG2*^S147A^ smRNA transfection led to rapid differentiation and maturation of OPCs (Fig. 3c, d) from three different hiPSC lines. Consistent with our previous data, on day 21 of differentiation, 3.4-fold (*p* < 0.001) and 2.6-fold (*p* < 0.003) increases in the mRNA expression levels of MBP and myelin oligodendrocyte glycoprotein (*MOG*), respectively, were observed in OLs transfected with *OLIG2*^S147A^ smRNA compared to those transfected with OLIG2^WT^ smRNA (qPCR, *n* = 3, Fig. 3e). This outcome was associated with a concurrent increase in the expression of other myelin-related genes, i.e., myelin-associated glycoprotein (*MAG*) (3.5-fold, *p* = 0.005, *n* = 3) and proteolipid protein 1 (*PLP1*) (3.2-fold, *p* = 0.039, *n* = 3, Fig. 3e). Together, these results indicated that *OLIG2*^WT^ smRNA-induced OPCs showed limited maturation, whereas *OLIG2*^S147A^ smRNA-induced OPCs were capable of maturing toward the terminal stage because critical myelin-related genes were expressed at high levels.

To further characterize MN generation during OL differentiation induced by smRNA, immunofluorescence staining of two MN-specific marker genes (β III-tubulin (TUJ1) and HB9) was performed in cells from the hiPSC1 line. As shown in Fig. 3f, g, a smaller proportion of hiPSC1-derived NPCs transfected with OLIG2^S147A^ smRNA were induced to differentiate into TUJ1- or HB9-positive MNs than those transfected with *OLIG2*^WT^ smRNA. Our results demonstrated that the *OLIG2*^S147A^ smRNA inhibited MN production and might have triggered the MN-OL fate switch of hiPSC-derived NPCs.

### *OLIG2* smRNA-induced OLs generate a multilayer myelin sheath in the brains of mice with cuprizone-induced demyelination

**Fig. 4:**
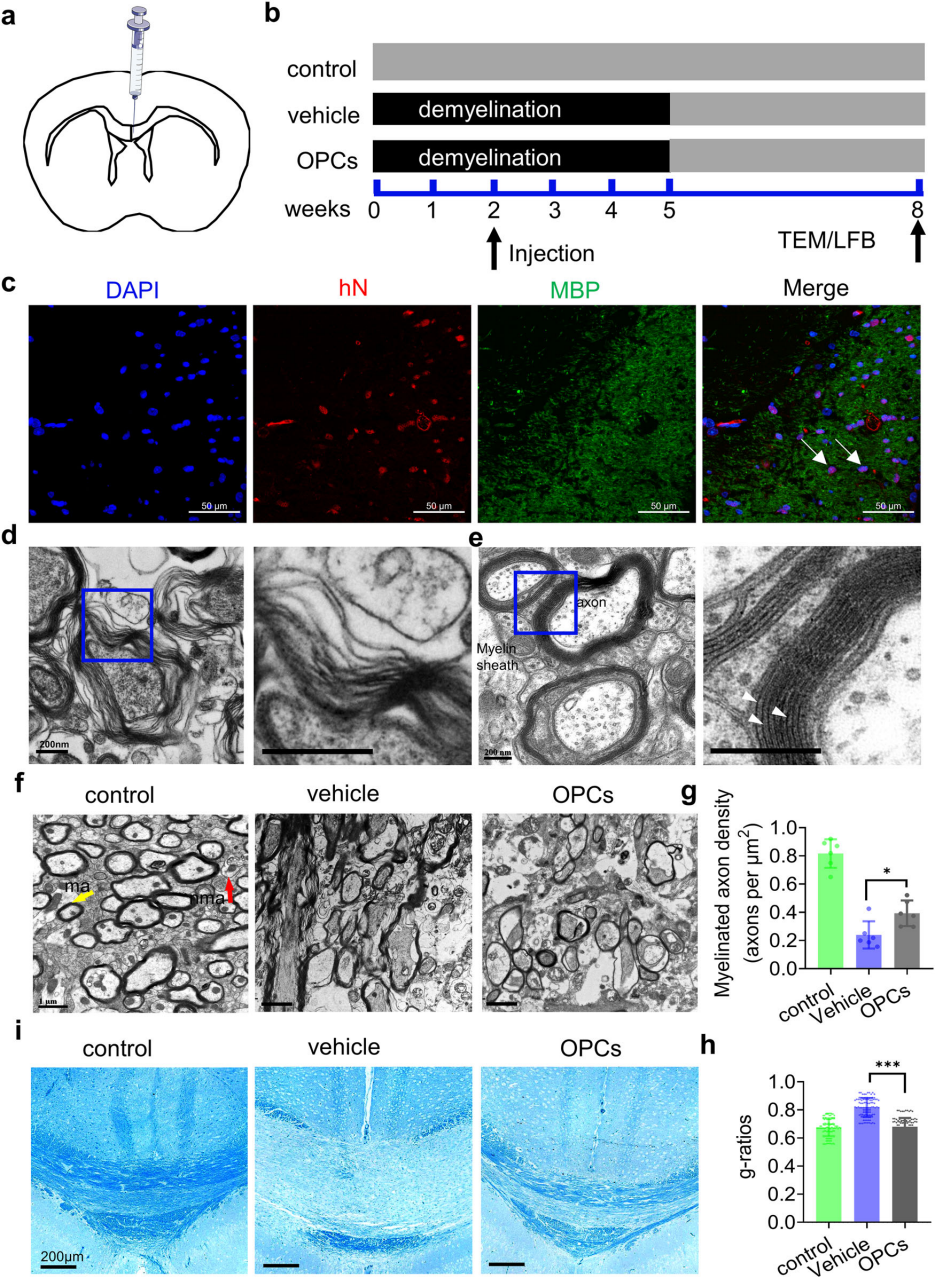
smRNA-induced OPCs give rise to functional myelin following transplantation in the brains of mice with cuprizone-induced demyelination. **a** Diagram showing the transplantation site in the corpus callosum. **b** Schematic of the mouse demyelination model and cell transplantation. Female C57BL/6 mice were provided a diet containing 0.2% cuprizone for 5 weeks. After demyelination was induced for 2 weeks, purified *OLIG2*^S147A^ smRNA-transfected O4^+^ OPCs (10^5^) were injected into the demyelinated mouse brain. **c** Representative images of immunofluorescence staining for hN and MBP. Transplantation of human OPCs (hN^+^ cells) into the corpus callosum of mice resulted in extensive generation of MBP^+^ OLs (green) by the human cells and positive staining for the human nucleus marker hN (red) for 8 weeks; scale bar, 20 μm. **d** Electron micrographs showing that cuprizone induced myelin disintegration and shedding in the mouse brain; scale bar, 200 nm. **e** Electron micrographs of myelinated axons exhibiting characteristic compact myelin with alternating major dense (arrowheads) and intraperiod lines; scale bar, 200 nm. **f** Low-magnification electron micrographs showing a portion of the corpus callosum in animals in the control, vehicle and OPC transplantation groups. Myelin sheath (yellow arrowhead, ma); lack of a compact myelin sheath (red arrowhead, nma). Scale bars, 2 μm. **g** The density of myelinated axons in each group (*n* = 6; *p < 0.05 using one-way ANOVA test). **h** Mean g-ratios of the control, vehicle and OPC groups. The myelin g-ratio was calculated as the ratio of the inner to the outer radius of the myelin sheath for a circular axon cross section (*n* = 100, ***p < 0.001, using one-way ANOVA test). **i** Representative images of the corpus callosum region stained with LFB after cuprizone administration and OPC transplantation; scale bar, 200 μm.

To assess myelination in in vivo, FACS-purified *OLIG2*^S147A^ smRNA-induced O4^+^ cells (purity > 92%, Fig. S5a) frozen in liquid nitrogen were resuscitated and cultured until they reached a suitable state for transplantation. The cells were stained with an antibody against O4 and displayed a distinct, typical branched morphology of mature OPCs or pre-OLs (Fig. S5b). They were injected into the corpus callosum of mice with cuprizone-induced demyelination, and mouse brain slices were obtained (Fig. 4a, b). Immunohistochemistry revealed efficient engraftment, as indicated by the presence of human hN^+^ cells coexpressing MBP in brain tissue 6 weeks after transplantation (Fig. 4c). We next sought to investigate the formation of a compact myelin ultrastructure around mouse axons by *OLIG2*^S147A^ smRNA-induced OLs in vivo. TEM of corpus callosum samples from mice with cuprizone-induced demyelination that had received vehicle or *OLIG2*^S147A^ smRNA-induced O4^+^ OPCs was performed (*n* = 5). We found that significant demyelination and disintegration occurred in mice from the vehicle group (Fig. 4d); however, the corpus callosum of the cell-transplanted mice showed dense mature compact myelin, which was characterized by major concentrically organized dense lines (Fig. 4e) and interlaminar tight junctions. The density of myelinated axons in the OPC group was significantly higher than that in the vehicle group and was similar to that in the sham group (Fig. 4f, g). Remyelination by engrafted OPCs was further analyzed and quantified by calculating the g-ratio, which is the ratio of the axon diameter to the total diameter of a myelinated fiber^30^. Compared to the vehicle group, the OPC group showed significantly lower g-ratio values (Fig. 4h). LFB staining showed spontaneous remyelination in the OPC group (Fig. 4i). These results indicated that *OLIG2* smRNA-induced OLs matured into myelin-forming cells and enhanced the remyelination process in the demyelinated mouse brain.

### The HSP70 complex binds to and promotes the function of OLIG2 in driving OL differentiation

**Fig. 5:**
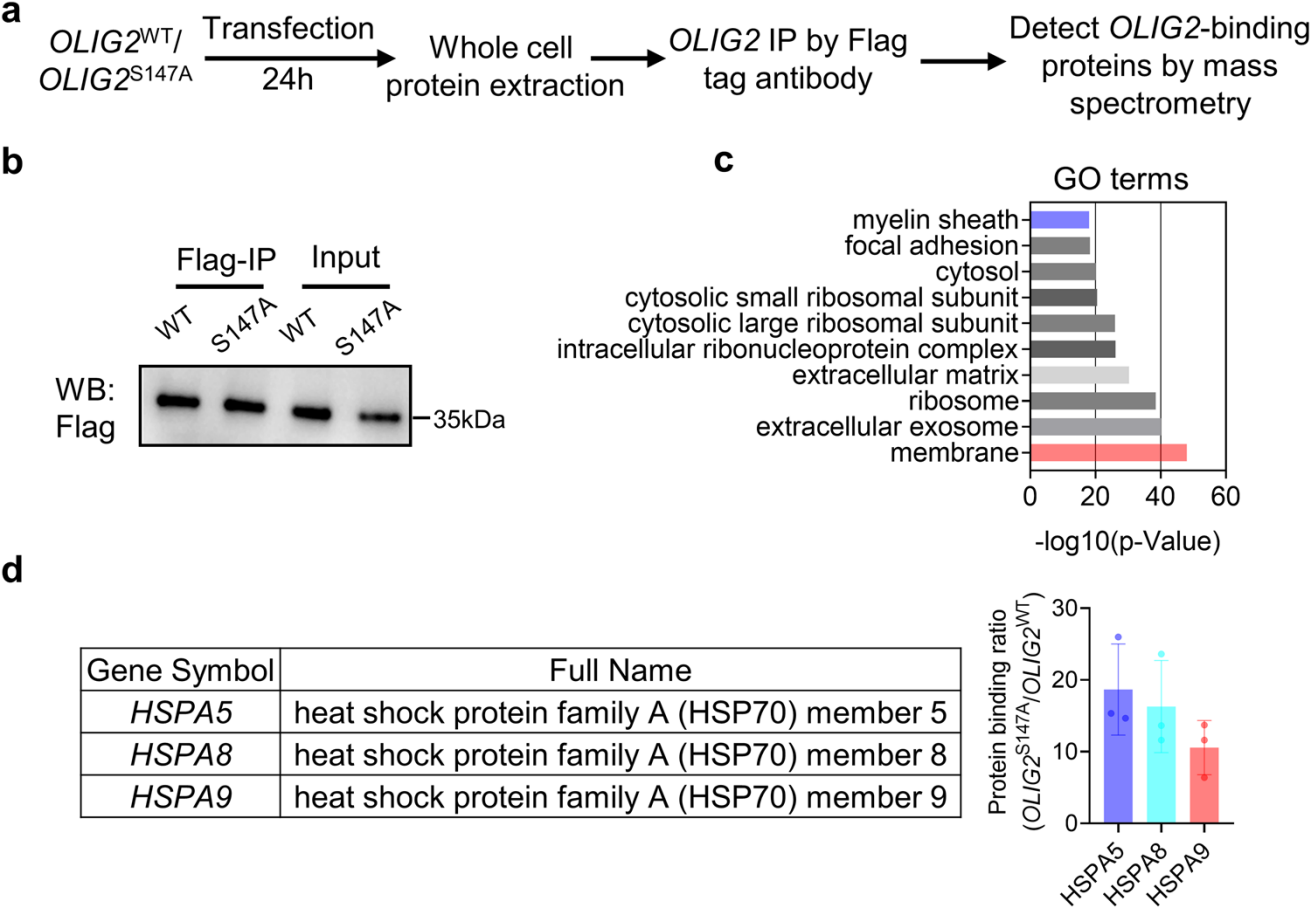
Proteomic analysis identified proteins that differentially bound to OLIG2^S147A^ and OLIG2^WT^ smRNA. **a** Schematic of the proteomic analysis protocol using hiPSC1-derived NPCs transfected with *OLIG2*^S147A^ or *OLIG2*^WT^ smRNA. **b** Western blot analysis of the *OLIG2*^S147A^ and *OLIG2*^WT^ proteins pulled down with a Flag tag. **c** Proteins exhibiting differential binding to *OLIG2*^S147A^ and *OLIG2*^WT^ were analyzed using the DAVID bioinformatics database to identify proteins involved in the myelin sheath pathway. **d** The *OLIG2*^S147A^/*OLIG2*^WT^ binding ratio of HSP70 complex components (HSPA5, HSPA8 and HSPA9) involved in the myelin sheath pathway.

We performed proteomic analysis to identify proteins that bind *OLIG2*^S147A^ and compare them with those that bind *OLIG2*^WT^, confirm the underlying mechanism of OL differentiation mediated by *OLIG2* smRNA, and determine the potential partners of *OLIG2* in promoting OPC/OL generation. As shown in Fig. 5a, we performed Flag IP by pulling down Flag-tagged *OLIG2*^WT^ and *OLIG2*^S147A^ and their binding proteins from NPCs 24 h after transfection with either *OLIG2*^WT^ or *OLIG2*^S147A^ smRNA. (Fig. 5b). Proteomic analysis led to the identification of 67 differentially expressed proteins in NPCs transfected with either *OLIG2*^WT^ or *OLIG2*^S147A^ smRNA (Supplementary Data 1). These differentially binding proteins were subjected to pathway enrichment analysis using the DAVID bioinformatics database (Supplementary Data 2). They were found to be enriched in the myelin sheath (Fig. 5c) and to be possible positive modulators of *OLIG2* transcriptional activity that drive more efficient OL differentiation. Among these elevated proteins that were enriched in the myelin sheath, proteins associated with *OLIG2*^S147A^ were found to include multiple chaperones, particularly three main members of the HSP70 complex (HSPA5, HSPA8, and HSPA9) (Fig. 5d), indicating a potential role for the HSP70 complex in regulating OLIG2-driven OL differentiation.

**Fig. 6:**
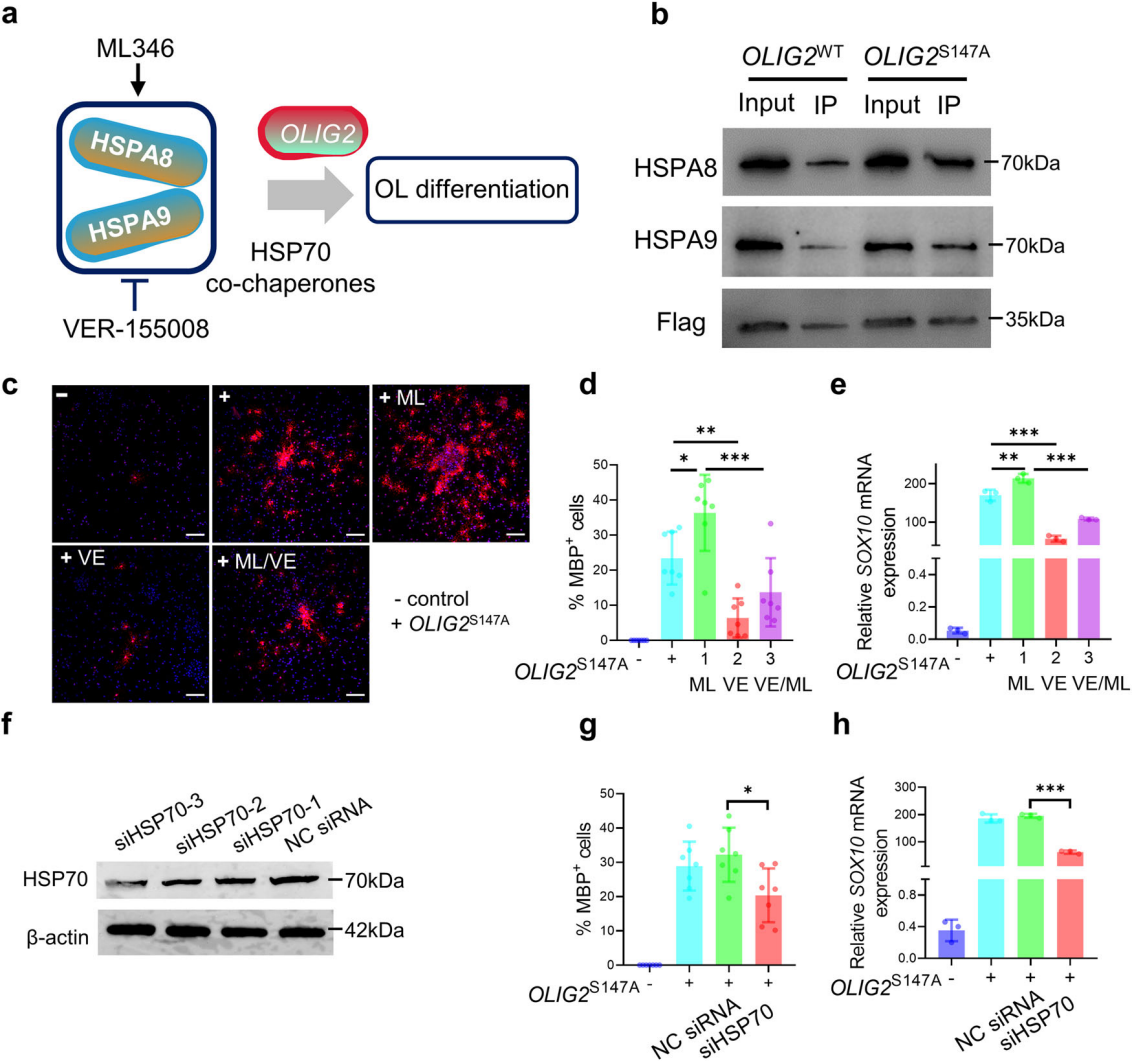
The HSP70 complex binds to *OLIG2* and promotes *OLIG2*-driven smRNA-induced OL differentiation. **a** Diagram of the HSP70 complex and our working model. Two main nuclear components of HSP70 were identified in our study. The HSP70 agonist VE and antagonist ML were utilized to induce and inhibit HSP70, respectively. **b** hiPSC1-derived NPCs were transfected with *OLIG2*^WT^ or OLIG2^S147A^ smRNA for 24 h, and nuclear proteins were extracted for Flag IP. The IP samples were immunoblotted to assess the levels of components of the HSP70 complex. **c** VE and ML were administered along with OLIG2^S147A^ smRNA during OL differentiation. MBP-positive OLs were detected on day 21 of differentiation; scale bar, 100 μm. **d** Quantification of the percentage of MBP-positive cells in the cultures (*n* = 7; *p < 0.05, **p < 0.01, and ***p < 0.001, using one-way ANOVA tests). **e** hiPSC1-derived NPCs underwent 6 daily transfections of OLIG2^S147A^ mRNAs and treated with VE and ML. At 24 h after transfection, the mRNA expression of *SOX10* was quantified by qPCR (*n* = 3; **p < 0.05, ***p < 0.001 using using one-way ANOVA test). **f** Knockdown of HSP70 in hiPSC1-derived NPCs. **g** Quantification of the percentage of MBP-positive cells in the cultures (*n* = 7; *p < 0.05 using one-way ANOVA test). hiPSC1-derived NPCs were transfected with siHSP70 along with *OLIG2*^S147A^ smRNA, OL differentiation was induced, and MBP-positive OLs were detected on day 21 of differentiation. **h** qPCR analysis of *SOX10* mRNA expression levels. hiPSC1-derived NPCs were transfected with siHSP70 along with OLIG2^S147A^ smRNA, and OL differentiation was then induced. At 24 h after transfection, *SOX10* mRNA expression was quantified by qPCR (*n* = 3; ***p < 0.001, using one-way ANOVA test).

Total protein was extracted from NPCs 24 h after transfection of either *OLIG2*^WT^ or *OLIG2*^S147A^ smRNA to validate the identities of the TF OLIG2-binding proteins. IP and immunoblot analyses were performed to validate the binding of the two candidate proteins HSPA8 and HSPA9, which are mainly located in the nucleus and cytosol, to *OLIG2*^31^. Indeed, based on our working model (Fig. 6a), HSPA8 and HSPA9 were consistently more strongly associated with *OLIG2*^S147A^ than with *OLIG2*^WT^ (Fig. 6b). Next, the HSP70 complex agonist ML346 (ML) and antagonist VER-1555008 (VE) were utilized to determine whether the efficiency of OL generation was regulated by HSP70 activity. ML acts as an activator of HSP70 expression by increasing HSF-1 activity^32^. ML treatment increased the yield of MBP-positive OLs by 45% following *OLIG2*^S147A^ smRNA treatment (Fig. 6c, d), suggesting that the HSP70 complex is a positive modulator of *OLIG2*^S147A^ activity. In contrast, VE is a functional HSC70 inhibitor that competitively binds to the ATP-binding pocket of HSP70^33,34^. As shown in Fig. 6d, the yield of MBP-positive OLs was significantly decreased after VE treatment, and VE also attenuated the effect of ML in promoting OL production. Furthermore, we determined the transcriptional activity of *OLIG2* by measuring the mRNA expression level of the *OLIG2* target gene *SOX10* in response to *OLIG2*^S147A^ smRNA transfection and ML and/or VE treatment (Fig. 6e). ML treatment significantly upregulated *SOX10* expression following transfection of *OLIG2*^S147A^ smRNA, while VE treatment attenuated this induction of *SOX10* expression and the effect of ML in promoting *SOX10* upregulation. We validated these findings by investigating the effect of genetic elimination of HSP70 factors on OL differentiation. To this end, three HSP70 siRNAs were used to knockdown HSP70 expression in hiPSC-derived NPCs (Fig. 6f). We found that the siRNA targeting HSP70 decreased MBP-positive OL generation after *OLIG2*^S147A^ smRNA transfection (Fig. 6g). Additionally, HSP70 siRNA significantly upregulated *SOX10* expression (Fig. 6h). Taken together, these results indicated that these two HSP70-modulating compounds may have regulated OL generation by affecting the transcriptional activity of *OLIG2*^S147A^.

## Discussion

smRNA-driven differentiation strategies for generating hiPSCs or hiPSC-derived specific cell types have been well documented in recent decades^10,11,35^. In this study, an smRNA encoding a TF was introduced into cells through nongenome-integrating viruses. This approach not only increased the expression level of the TF but also prevented the risk of tumorigenicity caused by genome integration. Here, we report for the first time that an smRNA-based differentiation strategy induced highly efficient generation of human OL cells from hiPSC-derived NPCs, while *OLIG2* smRNA failed to directly reprogram hiPSCs to OLs. We show that repeated transfection of *OLIG2* smRNA containing a phosphorylation site modification significantly enhanced the efficiency of OL generation from NPCs derived from hiPSCs.

Proteins are important functional molecules that maintain normal life activities. PTM of proteins is an important way to regulate the structure, localization, stability, and degradation rate of proteins. Previous studies have reported that protein phosphorylation promotes protein degradation in most cases^36,37^. Protein expression after *OLIG2*^S147A^ smRNA transfection was much higher than that after *OLIG2*^WT^ smRNA transfection, as shown in Fig. 3d. We speculate that inhibiting the phosphorylation of OLIG2 at serine 147 may delay the degradation of OLIG2 and improve the stability of OLIG2 expression. To the best of our knowledge, although some TFs or TF cocktails have been identified as key factors in OL development that depend on the integration of retroviral vectors^5,14,16–18^, OL fate conversion through *OLIG2* activation only has not been achieved. smRNA-based gene delivery has been shown to be highly efficient and safe for cell fate reprogramming^38–41^. A major advantage of the single *OLIG2* smRNA-based approach described in this article is the ability to design and modify the mRNA translation sequence to promote protein expression and transcriptional activity. This strategy is crucial to regulate the developmental fate switch of NPCs from MNs to OLs and to generate large numbers of functional OLs from hiPSCs. Li et al. reported that the TF *OLIG2* participates in MN-OL fate switching and is dephosphorylated at the onset of OL genesis. Replacing serine 147 with an alanine residue (S147A) has been shown to abolish MN production without preventing OL production in transgenic mice and chicks and cultured P19 cells^24^. Apparently, by controlling PTM of the *OLIG2* protein, such as phosphorylation, the function of *OLIG2* in promoting MN generation in the early neurogenesis stage can be eliminated^21^. Indeed, our results confirmed that *OLIG2* smRNA with a phosphorylation site modification downregulated the expression of the MN-related genes *NGN2* and *HB9* and enhanced the expression of *OLIG2* and functional O4^+^ OL generation. These findings are consistent with those reported in other publications. For example, Xue et al. reported that a phosphorylation site modification in proneural Atoh1 and Ngn2 effectively enhanced lineage-specific neuron generation from hiPSCs^35^. Proper PTM results in more precise localization and higher translational activity. Therefore, smRNA-induced OLIG2 expression is essential for MN-OL fate switching, and *OLIG2* smRNA delivery is a practical strategy for inducing OL differentiation from hiPSCs.

Our understanding of demyelinating diseases and the development of new therapeutic options are hampered by the limited ability to obtain stable human OLs. As demonstrated here, *OLIG2* smRNA-driven OL differentiation from hiPSCs is very robust, resulting in the production of up to 70% O4^+^ OPCs. Similar to OLs generated by other protocols, these smRNA-induced OPCs mature into OLs in vitro, indicating that they may promote remyelination in the CNS after demyelination. Although we observed that transplanted smRNA-induced OPCs survived in the mouse brain and eventually differentiated into myelinating OLs, remyelination might not have resulted exclusively from the direct effect of donor-derived OPCs. Indeed, OPCs have been shown to directly regulate myelin sheath formation in the CNS. In addition, exosome-mediated molecular signals from OPCs may also play an important role in remyelination after myelin injury, but corresponding in-depth studies are lacking. Exosomes are membranous vesicles of nanometer size (30–100 nm in diameter) that are secreted by many types of cells, such as glial cells and immune cells^42^. Exosomes contain mRNAs, microRNAs (miRs), various proteins (such as Alix and HSP70), and signaling molecules (CD9 and CD63) that are critical mediators of intercellular signal transduction. For example, exosomes secreted from bone marrow mesenchymal stromal cells (MSCs) exert a significant therapeutic effect on enhancing remyelination and reducing neuroinflammation in the demyelinated CNS^43^. Therefore, the bystander effect of remyelination induced by infused OPCs in mice with cuprizone-induced demyelination should not be ignored. Herein, we propose that exosome-derived infused OPCs may act on resident OPCs, indirectly and partially promoting remyelination upon OPC transplantation.

Reprogramming of hiPSCs into OPCs/OLs largely relies on regulators that modulate the stability and/or transcriptional activity of the OLIG2 protein. Our proteomic analysis identified *OLIG2*-binding proteins in hiPSC-derived NPCs for the first time. *OLIG2*^S147A^ and *OLIG2*^WT^ showed differential binding affinity for a set of binding partners. Poly (ADP-ribosyl) polymerase-1 (PARP-1) has been identified as a positive modulator of OL differentiation and remyelination^44–47^. Previous studies performed in our laboratory also revealed that PARP-1 acts as an intrinsic driver of OL development and myelination by stabilizing myelin mRNA translation. Specific inhibition of PARP1-mediated PARylation activity in OLIG2-expressing cells further compromises OL differentiation and CNS remyelination^32^. Indeed, consistent with our proteomic data, the level of PARP-1 bound to *OLIG2*^S147A^ was ~ 15-fold higher than that bound to *OLIG2*^WT^ (Fig. S6). Therefore, PARP-1 might be a crucial factor that mediates OLIG2 PTM involved in OL differentiation. Moreover, the increase in PARP-1 binding to *OLIG2*^S147A^ was consistent with the elevated expression of downstream myelin-associated genes, such as *MBP*, *MOG*, *MAG* and *PLP1*, as shown in Fig. 3e. The higher binding affinity of *OLIG2*^S147A^ for PARP-1 than *OLIG2*^WT^ indicated a potential mechanism by which *OLIG2*^S147A^ promotes differentiation ability, but these molecular events remain to be further studied in detail. Most importantly, in the present study, we found that the HSP70 complex might stabilize OLIG2 and lead to more robust oligodendroglia lineage-specific gene induction (particularly at the bulk level). We show, for the first time, that the HSP70 complex is a functional *OLIG2*-binding partner and that the HSP70 agonist ML promotes OL generation. The HSP70 complex is widely known to function as a chaperone that supports the folding of diverse target proteins^48,49^. Several studies have shown that constitutively expressed HSP70 is required for the optimal expression of MBP during OL differentiation^50^. Our study revealed a novel function for the HSP70 complex in promoting the transcriptional activity of *OLIG2* and its function in OL lineage specification. Our findings provide an entirely new perception and understanding of the structural basis of the OLIG2/HSP70 interaction to optimize OL production from NPC-derived hiPSCs.

Next, several important limitations of this smRNA-driven strategy are discussed. First, it was unlikely to directly induce OPC differentiation from uncommitted hiPSCs. Indeed, according to our data, forced expression of *OLIG2* smRNA alone did not result in the generation of NG2- or PDGFRα-positive cells; in contrast, only neural-committed NPCs were observed. This outcome was consistent with previous findings showing that the expression of the single TF SOX10 alone does not directly induce an OL fate of hiPSCs^5,51^. Recently, Chanoumidou et al. published a rapid protocol for the generation of O4^+^ cells from human fibroblasts induced by ectopic expression of a TF cocktail including SOX10, OLIG2, and NKX6.2 and showed that the fibroblasts further differentiated into MBP^+^ mature OL-like cells within 16 days^52^. TF cocktails show a powerful propensity for inducing OL lineage specification. *OLIG2*^WT^ smRNA showed limited ability to achieve OL specification of hiPSCs, partially due to the lack of certain partner proteins. It is logical to assume that other essential TF smRNAs that can directly generate OLs from hiPSCs or even human fibroblasts will be identified in future studies. Another limitation of this study involves the transplantation experiment; it is unclear whether hiPSC-derived O4^+^ cells also possess the ability to promote myelin regeneration in vivo. Although we validated the effectiveness of our smRNA-driven OL differentiation strategy in a variety of hiPSC lines, the presented animal data could be hiPSC1 line-specific, as the findings were not proven for any other hiPSC lines. Furthermore, it is insufficient to focus only on the effect of OPCs in demyelinating diseases. hiPSC-derived OPC transplantation has also shown significant therapeutic potential in other human brain diseases, such as ischemic stroke. Therefore, it is very meaningful to broaden the application scope of smRNA-induced OPCs and further confirm the efficacy of OPC transplantation in other disease models.

In summary, highly efficient hiPSC differentiation strategies are critical for the application of hiPSC technology in regenerative medicine. Traditional methods based on chemical compounds and viruses are relatively slow, variable, or unsafe. In this study, a highly efficient strategy was established based on using a smRNA encoding the single transcription factor *OLIG2* with dephosphorylation modification to drive rapid hiPSC differentiation into functional OLs. These discoveries will facilitate the applications of hiPSC-derived OLs in demyelinating disease modeling and therapy and guide the development of robust methods for generating various lineage-specific progeny from hiPSCs.

## Methods

### smRNA synthesis and cell transfection

The coding sequences of human *OLIG2*^WT^ and *OLIG2*^S147A^ were cloned into a vector containing the T7 promoter and poly(A) tail for IVT. The open reading frames (ORFs) of *OLIG2* were cloned into the PCR2-UTR-R1R2 vector according to the LR reaction protocol (Thermo Fisher Scientific, Waltham, MA, USA). The PCR2-UTR-B1B2 vector was linearized with a restriction enzyme that cuts outside the ORF. The linearized vector was used as the template for tail PCR utilizing a 5′ primer with a T7 polymerase promoter sequence and a 3′ UTR- directed primer with a long poly T tail. The PCR product was gel purified and used as a template for an IVT protocol that was performed essentially as described by Mandal and Rossi. The IVT reaction included an anti-reverse cap analog (ARCA, B8175, APExBIO, Houston, USA) and the modified nucleotides 5-methylcytidine-5′-triphosphate and pseudouridine-5′-triphosphate, to reduce cytotoxicity due to the activation of innate immune responses. The purified smRNA was verified and stored as aliquots at −80 °C until use (Table S1). In each well of a 12-well plate, we incubated smRNAs with 1.5 μL of ScreenFect transfection reagent in 75 μL of transfection buffer for 15 min before adding the mixture to cultured cells. Cell proliferation and death were analyzed using the Cell Counting Kit-8 (CCK-8) assay kit (CK101-01, Data Inventory Biotechnology, Hong Kong, China) according to the manufacturer’s instructions.

### hiPSC culture and neural differentiation

hiPSC lines (hiPSC-UC17P3-C5P9, hiPSC-UCF3P2-C3P3, and hiPSC-UCM5P3-C5P4; referred to as hiPSC1, hiPSC2, and hiPSC3, respectively) were obtained from the Guangzhou Institutes of Biomedicine and Health Chinese Academy of Science (Guangzhou, China). All three hiPSC lines in this study were derived from normal human urine epithelial cells, and cytogenetic analysis of all hiPSC lines showed a normal karyotype (Fig. S7). For growth under feeder-free conditions, hiPSCs were cultured in Matrigel-coated 12-well plates in mTeSR medium (#85850, Stem Cell Technologies, Canada) medium. The cells were incubated at 37 °C in 5% CO_2_, and were mechanically split every 5 days at 1:8 ratios using ReLeSR (Stem Cell Technologies, Canada) according to the methods described by the manufacturer.

For the differentiation of hiPSCs into NPCs. Briefly, passages 20 to 30 hiPSCs were collected and enzymatically and mechanically dissociated. The diluted colonies were replated in a six-well plate. Once the confluence of hiPSCs reached about 20%, Culture medium was then switched to Neural Induction Medium 1 for 2 days (NIM1: 50% DMEM/F12, 50% Neurobasal, 1X B27, 1X N2, 1X GlutaMAX and 10 ng/mL hLIF, 4 µM CHIR99021, 3 µM SB431542, 2 µM Dorsomorphin, and 0.1 µM Compound E. Next, the culture medium was changed to NIM2 for another 5 days (NIM2: 50% DMEM/F12, 50% Neurobasal, 1X B27, 1X N2, 1X GlutaMAX and 10 ng/mL hLIF, 4 µM CHIR99021, 3 µM SB431542, and 0.1 µM Compound E). At day 8, single cell passage was performed with ACCUTASE, and the medium was switched to neural stem cell maintenance medium (NSMM: 1X B27, 1X N2, 1X GlutaMAX, 10 ng/mL hLIF, 3 µM CHIR99021 and 2 µM SB431542. Y-27632 (M1817, Abmole, USA) with a final concentration of 10 µM was added to the culture medium for each passage. To prevent cell differentiation, culture medium should be changed every day during the culture of hiPSCs and NPCs. For the detailed composition of the media used in this experiment, see Table S2.

### OPC differentiation

For OL lineage differentiation, a two-step differentiation protocol was utilized. hiPSC-derived NPCs were seeded at a density of 1.5 × 10^5^ cells per well in a 12- well plate coated with poly-L-ornithine/laminin (Sigma‒Aldrich, MO USA). NPCs were transfected with *OLIG2* ^WT^ or *OLIG2* ^S147A^ smRNA using ScreenFect transfection reagent (293-75901, Wako, Japan) for 6 days, and the culture medium was changed to glial induction medium (GIM) for 4 days. Then, the medium was replaced with differentiation medium (DM). A detailed description of the GIM and DM is provided in Table S2. The cells were also cryopreserved in medium containing 40% neurobasal medium with B27 supplement, 50% fetal bovine serum (DIB-12B-10X50ML, Data Inventory Biotech, China) and 10% DMSO.

### Immunofluorescence staining and immunohistochemical staining

Cells on coverslips were fixed with 4% paraformaldehyde for 15 min at room temperature, and washed with PBS three times. After fixation, cells were then blocked with 4% bovine serum albumin (BSA, A8010, Solarbio, China) for 30 min at 37 °C and then incubated with an appropriate primary antibody overnight. The next day, the coverslips were incubated with fluorescent dye-conjugated secondary antibodies (Invitrogen, San Diego, CA, USA) for 1 h at room temperature in the dark. The cells were washed three times with PBS counterstained with 4′,6-diamidino-2-phenylindole (Vector Laboratories, Burlingame, CA, USA) for at least 10 min, washed with PBS three times, and mounted with mounting medium (Invitrogen).

For immunohistochemical staining of mouse brain samples, after the paraffin sections were dewaxed, they were placed in 0.1 mol/L citric acid repair solution (pH 6.0) for antigen retrieval. Then, they were permeabilized with 0.2% Triton and blocked with 3% BSA solution at 37 °C for 30 min. The sections were incubated with primary antibody overnight at 4 °C and then with secondary antibody for 1 h the next day. After that, tissue autofluorescence was eliminated with a kit (Vectorlabs, Vector True View Autofluorescence Quenching Kit), and then the nucleus was counterstained with DAPI. Finally, anti-fluorescence quenching agent was added for sealing. All coverslips and mouse brain samples were imaged using a fluorescence microscope (Eclipse Ti2-U, Nikon, Japan). The fluorescence intensity and proportion of positive cells were quantified by ImageJ software (NIH, USA). All primary antibodies used in this study are listed in Table S3. Paraffin-embedded coronal brain sections were stained with luxol fast blue (LFB, G1030, Servicebio, China) to examine demyelination.

### Immunoblotting

Whole-cell lysates or nuclear protein lysates were extracted, and then the concentration was determined with a BCA protein assay kit (Thermo Fisher Scientific, USA). Fifty micrograms of total protein were loaded into each well of a 10% SDS–PAGE gel and separated by electrophoresis. The primary antibodies are listed in Table S3. After incubation with primary antibodies, the membranes were incubated with secondary antibodies labeled with HRP (Solarbio, China), and protein expression was normalized to the expression of the housekeeping protein tubulin in the same sample.

### Quantitative real-time PCR (qPCR)

Total RNA was extracted using an EZ-Press RNA Purification Kit (B0004D, EZBioscience, Roseville, USA), and the RNA concentration was measured by a Nanodrop spectrophotometer (Thermo Scientific). cDNA was subsequently synthesized using an EZscript All-in-one Reverse Transcription Kit (RT3P, EZBioscience). qPCR was performed using Real Time PCR EasyTM-SYBR Green I (QP-01014, Foregene, China). The primer sequences used for qPCR are provided in Table S4 (Tsingke, China).

### Immunoprecipitation (IP)

Twenty-four hours after smRNA transfection, whole-cell lysates were extracted, and the protein concentration was determined with a BCA protein assay kit (Thermo Fisher Scientific, USA). Ten micrograms of primary antibody were covalently cross-linked to 25 μL of protein A/G magnetic beads (L00277, GenScript). A portion of each sample was saved as input. An equal amount (1 mg) of each protein extract was incubated with Protein A/G magnetic beads cross-linked with anti-Flag antibody (AP1013A, Abcepta, USA) overnight at 4 °C. The Pierce Protein A/G magnetic beads were washed three times using Pierce IP lysis/wash buffer before LC–MS/MS analysis.

### LC–MS/MS

Aliquots of proteins were mixed with 200 μL of 8 M urea in Nanosep centrifugal DEVICES (Pall). The devices were centrifuged at 12,000 × *g* for 20 min at 20 °C. All subsequent centrifugation steps were performed under the same conditions to allow maximal concentration. Then, 200 μL of an 8 M urea solution supplemented with 10 mM DTT was added, and the reduction reaction was performed for 2 h at 37 °C. The solution was removed by centrifugation, and 200 μL of an 8 M urea solution supplemented with 50 mM iodoacetamide (IAA) was added. The sample was incubated in the dark for 15 min at room temperature. The ultrafraction tube was washed with 200 μL of 8 M urea three times and 200 μL of 25 mM ammonium bicarbonate three times by centrifugation at 12,000 × *g* for 20 min at room temperature. Then, 100 μL of 25 mM ammonium bicarbonate containing 0.01 μg/μL trypsin was added to each filter tube. The tubes were incubated at 37 °C for 12 h and washed twice with 100 μL of 25 mM ammonium bicarbonate by centrifugation at 12,000 × *g* for 10 min. The flow-through fractions were collected and lyophilized. The lyophilized peptide fractions were resuspended in ddH_2_O containing 0.1% formic acid, and 2 μL aliquots were loaded into a nanoViper C18 (Acclaim PepMap 100, 75 μm × 2 cm) trap column. Online chromatography separation was performed using an Easy nLC 1200 system. The trapping and desalting procedures were conducted with 20 μL 100% solvent A (0.1% formic acid). Then, an elution gradient of 5-38% solvent B (80% acetonitrile, 0.1% formic acid) was used to elute peptides from an analytical column (Acclaim PepMap RSLC, 75 μm × 25 cm C18-2 μm 100 Å) over 120 min. Data-dependent acquisition (DDA) mass spectrum techniques were used to acquire tandem MS data on a Thermo Fisher Q Exactive mass spectrometer (Thermo Fisher Scientific, USA) fitted with a Nano Flex ion source. Data were acquired using an ion spray voltage of 1.9 kV and an interface heater temperature of 275 °C. For a full mass spectrometry survey scan, the target value was 3 × 10^6^, and the scan ranged from 350 to 2000 m/z at a resolution of 70,000 and a maximum injection time of 100 ms. For the MS2 scan, only spectra with a charge state of 2–5 were selected for fragmentation by higher-energy collision dissociation with a normalized collision energy of 28. The MS2 spectra were acquired in the ion trap in rapid mode with an AGC target of 8000 and a maximum injection time of 50 ms. Dynamic exclusion was set for 25 s. The MS/MS data were analyzed for protein identification and quantification using PEAKS Studio 8.5 software. The local false discovery rate at PSM was 1.0% after searching the target database with a maximum of two missed cleavages. Oxidation (M), acetylation (protein N-term), deamination (NQ), Pyro-glu from E and Pyro-glu from Q were selected as variable modifications, and carbamidomethylation of cysteine was selected as a fixed modification. The precursor and fragment mass tolerances were set to 10 ppm and 0.05 Da. The label-free quantitative parameter was set to a fold change of 1.2.

### Fluorescence-activated cell sorting (FACS) and cell cryopreservation

The induction cultures were maintained for 21 days in DM, and O4^+^ cell sorting was performed with a BD FACSAria cell sorter (BD Biosciences, San Jose, CA, USA) on day 21 of differentiation as described previously following the manufacturer’s instructions. Negative controls were used to set gates following doublet discrimination. Purified O4^+^ cells were cryopreserved until further cell transplantation. Purity was checked in all cases by flow cytometry, and > 92% O4^+^ cell purity was obtained for the isolated population. For cell cryopreservation, the purified O4^+^ fraction was resuspended in OL DM and mixed 1:1 with ice-cold Pro-Freeze medium (Lonza, USA) containing 15% DMSO. The cells were immediately stored in a freezing container at −80 °C overnight and transferred the next day to liquid nitrogen for long-term storage.

### Flow cytometry analysis

Cells were enzymatically harvested using Accutase, centrifuged and resuspended in 100 μL of FACS buffer (1X PBS, 2% fetal bovine serum and 0.02% sodium azide). To determine NG2, PDGFRa, and O4 expression, *OLIG2* smRNA-induced progenies were incubated or not (as a control) with 1/20 dilutions of NG2-PE (C06035P, Signalway antibody, USA), PDGFRa-APC (323512, BioLegend, USA), and O4-APC (130-118-978, Miltenyi Biotec, Germany) antibodies or for 15 min at 4 °C, washed and resuspended in 150 μL of FACS buffer. The cells were analyzed using a BD flow cytometer (Beckman Coulter, CytoFLEXS). All flow cytometry data were further analyzed using BD CytoExpert software (BD Biosciences, USA).

### Transplantation of smRNA-induced OPCs in a cuprizone-induced demyelination mouse model

Eight- to ten-week-old female C57BL/6 mice (*n* = 5) were fed an AIN-93G purified rodent diet containing 0.2% cuprizone (Dyets, D200218) for 5 weeks; thereafter, the cuprizone-infused food was removed, and the mice were given a normal diet. Mice with demyelination induced by cuprizone serve as a well-proven model to test the functional capacity of transplanted OPCs to form MBP-positive compact myelin sheaths around axons. The demyelinated mice were injected with 100,000 *OLIG2*^S147A^ smRNA-induced O4^+^ OPCs into the corpus callosum using a Hamilton syringe (KW-ZSB, NJKEWBIO, China) at the following coordinates according to our previously published protocols^19^: 0.2 mm anteroposterior and 1.1 mm mediolateral to bregma and 2 mm dorsoventral from the skull surface. The control group included mice that were fed normal food for 5 weeks and underwent sham operation. The mice were intraperitoneally injected with 10 mg/kg cyclosporine daily for immune suppression beginning 2 days before cell transplantation. Postoperative care was provided according to Institutional Animal Care and Use Committee (IACUC) protocols approved by Sun Yat-sen University. The mice were sacrificed after 8 weeks, and corpus callosum sections (2–3 mm) containing the injection site were microdissected and processed for imaging under an electron microscope or for confocal imaging. The animal study protocol was approved by the Sun Yat-sen University Animal Use and Care Committee (SYSU-IACUC-2021-000776).

### Transmission electron microscopy

Brain samples were isolated from mice perfused with 4% paraformaldehyde, and then the corpus callosum was isolated and fixed with PBS containing 2.5% glutaraldehyde for 2 h. Next, the corpus callosum was washed, fixed with 1% osmium tetroxide, dehydrated in acetone, and embedded in epon. Afterward, 70–80 nm thin sagittal sections cut with a diamond knife were mounted on copper slot grids coated with Formvar. After staining with uranyl acetate and lead citrate, they were scanned with a JEM-1230 transmission electron microscope. Approximately 100 myelinated axons were measured for each group, and the g-ratios were calculated using ImageJ Pro software (NIH, USA).

### Construction of siRNAs and cell transfection

Three heat shock protein 70 (HSP70)-specific siRNAs targeting the human HSP70 sequence were synthesized as double-stranded oligonucleotides (MHBIO, China). hiPSC1-derived NPCs were seeded 24 h prior to transfection in 12-well plates. Cell transfection was conducted using Lipofectamine 2000 transfection reagent (DIB034, Data invention Biotech, China). Briefly, cells were incubated with HSP70 siRNAs (0.5 nM) or NC siRNA control in NSMM at 37 °C and 5% CO_2_ for 24 h. The culture medium was replaced with normal medium at the end of the incubation period. The efficacy of HSP70 knockdown was assessed after 2 days by western blotting. The sequences of the siRNAs designed in this study are provided in Table S5.

### Statistics and reproducibility

Prism™ 8.0.2 for Windows (GraphPad) was used for the statistical analysis. All least three independent replicates were performed for each experiment. The number of biological replicates “*n*” define the number of individual samples used per group for each experiment as indicted in the legends. The significance of differences between two measurements was determined by two-tailed unpaired Student’s *t* test, and the significance of differences among groups of three or more was determined by one-way analysis of variance (ANOVA) test. The data are presented as the mean ± standard error of the mean. Differences in mean values between different treatment groups were considered significant at *p* < 0.05 (presented as “ *”), *p* < 0.01 (presented as “ **”), and *p* < 0.001 (presented as “ ***”).

## Supplementary information

Supplementary Information

Description of Additional Supplementary Files

Supplementary Data 1

Supplementary Data 2

Supplementary Data 3

## Data Availability

The datasets used and/or analyzed during the current study are available from the corresponding authors upon reasonable request. The mass spectrometry proteomics data have been deposited to the ProteomeXchange Consortium via the PRIDE partner repository with the dataset identifier PXD036975 and 10.6019/PXD036975. Source data underlying the graphs is presented in Supplementary Data 3. Original and uncropped immunoblots are presented in Fig. S8.
