# Publisher Correction: Rapid differentiation of hiPSCs into functional oligodendrocytes using an *OLIG2* synthetic modified messenger RNA

**DOI:** 10.1038/s42003-022-04171-5

**Published:** 2022-11-03

**Authors:** Jian Xu, Zhihua Yang, Rui Wang, Fumei He, Rong Yan, Yidi Zhang, Liying Yu, Wenbin Deng, Yichu Nie

**Affiliations:** 1 School of Pharmaceutical Sciences (Shenzhen), Shenzhen Campus of Sun Yat-sen University, Shenzhen, 518107 China; 2 Stroke Center, The Fifth Affiliated Hospital of Guangzhou Medical University, Guangzhou, 510799 China; 3 Clinical Research Institute, The First People’s Hospital of Foshan, Foshan, 528000 China

**Keywords:** Induced pluripotent stem cells, Demyelinating diseases

Correction to: *Communications Biology* 10.1038/s42003-022-04043-y, published online 14 October 2022.

Supplementary Data 1 and 3 were missing from this article and have now been uploaded.

This has now been corrected in the HTML version of the Article.

## Supplementary information

Supplementary Data 1

Supplementary Data 3

