# Supplementary material for: Rapid differentiation of hiPSCs into functional oligodendrocytes using an OLIG2 synthetic modified messenger RNA
Source: Commun Biol. 2022 Oct 14;5:1095. doi: 10.1038/s42003-022-04043-y (PMC9568531; doi:10.1038/s42003-022-04043-y)
Supplement: Supplementary file 1 — Supplementary Information [file 42003_2022_4043_MOESM1_ESM.pdf]

Rapid differentiation from hiPSCs into functional oligodendrocytes using *OLIG2* synthetic modified messenger RNA

Jian Xu [1,*], Zhihua Yang [2,*], Rui Wang [3,*], Fumei He [1], Rong Yan [3], Yidi Zhang [1], Liying Yu [2], Wenbin Deng [1,#.] Yichu Nie [1,3,#]

1 School of Pharmaceutical Sciences (Shenzhen), Shenzhen Campus of Sun Yat-sen University, Shenzhen 518107, China; 2 Stroke Center, The Fifth Affiliated Hospital of Guangzhou Medical University, Guangzhou 510799, China; 3 Clinical Research Institute, The First People's Hospital of Foshan, Foshan 528000, China;

* These authors contributed equally to this work.

Supporting Information

Supplementary Tables

Table S1: The concentration and quality of smRNAs

smRNAs	concentration(μg/ul)	260/280	260/230
OLIG2^{S147A}	1.89	2.00	2.20
OLIG2^{WT}	2.44	1.98	2.18

Table S2. Detailed composition of culture medium

Media	Components	Provider	Final con.
**NIM1**	DMEM/F12	Life Technologies	50%
	Neurobasal	Gibco	50%
	B27 Supplement (50x)	Stem cell technology	1X
	N2 Supplement(100x)	Stem cell technology	1X
	Recombinant hLIF	PEPROTech	10 ng/mL
	SB431542	Med Chem Express	3 μM
	CHIR99021	Med Chem Express	4 μM
	GlutaMAX	Life Technologies	1X
	Compound E	Med Chem Express	0.1 μM
	Dorsomorphin	Med Chem Express	2 μM
**NIM2**	DMEM/F12	Life Technologies	50%
	Neurobasal	Gibco	50%
	B27 Supplement (50x)	Stem cell technology	1X
	N2 Supplement(100x)	Stem cell technology	1X
	Recombinant hLIF	PEPROTech	10 ng/mL
	SB431542	Med Chem Express	3 μM
	CHIR99021	Med Chem Express	4 μM
	GlutaMAX	Life Technologies	1X
	Compound E	Med Chem Express	0.1 μM
**NSMM**	DMEM/F12	Life Technologies	50%
	Neurobasal	Gibco	50%
	B27 Supplement (50x)	Stem cell technology	1X
	N2 Supplement(100x)	Stem cell technology	1X
	Recombinant hLIF	PEPROTech	10 ng/mL
	SB431542	Med Chem Express	2 μM
	CHIR99021	Med Chem Express	3 μM
	GlutaMAX	Life Technologies	1X

**GIM**	DMEM/F12	Gibco	
	B27 supplement lacking vitamin A	Stem cell technologies	1X
	N2 Supplement(100x)	Stem cell technologies	1X
	penicillin/streptomycin	Gibco	1%
	SAG	Med Chem Express	1 μM
	PDGF-AA	R&D Systems	10 ng/mL
	NT-3	Millipore	10 ng/mL
	IGF-I	R&D Systems	10 ng/mL
	AA	Sigma-Aldrich	200 μM
	T3	Sigma-Aldrich	60 ng/mL
**DM**	DMEM/F12	Gibco	
	B27 supplement lacking vitamin A	Stem cell technologies	1X
	N2 Supplement(100x)	Stem cell technologies	1X
	penicillin/streptomycin	Gibco	1%
	NT-3	Millipore	10 ng/mL
	IGF-I	R&D Systems	10 ng/mL
	AA	Sigma-Aldrich	200 μM
	T3	Sigma-Aldrich	60 ng/mL
	dbcAMP	Sigma-Aldrich	100 μM

Table S3. Primary antibodies used for immunostainings

Antigen	Dilution	Reference	
OLIG2	1/100	Rabbit IgG	Abcam ab254043
PAX6	1/200	Rabbit IgG	CST #60433
NESTIN	1/3200	Mouse IgG	CST #33475
O4	1/500	Mouse IgM	R&D MAB1326
O4-APC	1/50	Mouse IgG	Miltenyi Biotec 130-118-978
NANOG	1/100	Rabbit IgG	CST #4903S
SSEA4	1/500	Mouse IgG	CST #4755T
NG2	1/500	Rabbit IgG	Abcam ab129051
NG2-PE	1/20	Rabbit IgG	SAB C06035P
PDGFRa-APC	1/20	Rabbit IgG	Biolegend 323512
PDGFRa	1/1000	Rabbit IgG	CST #3174
MBP	1/50	Rabbit IgG	CST #78896
hN	1/200	Mouse IgG	Sigma-Aldrich MAB1281
Flag	1/1000	Rabbit IgG	Abcepta AP1013A
HSPA8	1/1000	Rabbit IgG	Abcepta AP2872a
HSPA9	1/1000	Rabbit IgG	Abcepta AP10160c
HSP70	1/1000	Rabbit IgG	Solarbio, K200048M
eGFP	1/100	Rabbit IgG	Abcam ab184601
SOX10	1/1000	Rabbit IgG	Abcam ab264405

Table S4. Sequence of primers for qPCR analysis.

Gene name	Species	Sequence of promers	
β-actin	Human	Forward	CCAGAGCCCGATGACCTTTTT
		Reserve	CACTGCCTCCTAGCTTGTCC
NKX2.2	Human	Forward	GAGGACGACGACGAATACAAC
		Reserve	GTTCGAGGGTTTGTGCTTCTT
SOX10	Human	Forward	CCTCACAGATCGCCTACACC
		Reserve	CATATAGGAGAAGGCCGAGTAGA
PLP1	Human	Forward	ACCTATGCCCTGACCGTTG
		Reserve	TGCTGGGGAAGGCAATAGACT
NGN2	Human	Forward	AGGAAGAGGACGTGTTAGTGC
		Reserve	GCAATCGTGTACCAGACCCAG
HB9	Human	Forward	CTCCTACTCGTACCCGCAG
		Reserve	TTGAAGTCGGGCATCTTAGGC
MBP	Human	Forward	GGCCGGACCCAAGATGAAAA
		Reserve	CCCCAGCTAAATCTGCTCAGG
MOG	Human	Forward	GGCAGCAATGGAATTGAAAGTAG
		Reserve	TGGGGTCCTAGAACACCAAAG
IFNA	Human	Forward	ACCCACAGCCTGGATAACAG
		Reserve	ACTGGTTGCCATCAAACTCC
IFNB	Human	Forward	CATTACCTGAAGGCCAAGGA
		Reserve	CAGCATCTGCTGGTTGAAGA
MAG	Human	Forward	GGTGTCTGGTACTTCAATAGCC
		Reserve	CTCTCGTGGACTACTTGGGTG

Table S5. Sequence of primers for HSP70 knockdown.

RNAi Name	Species Specificity	Target sequences
CEPT1 shRNA1	Human	5`- ACTGTAGCAGGGACCATATTT-3`
CEPT1 shRNA2	Human	5`- GGCACCTCTGTGGGCATATAT-3`
CEPT1 shRNA3	Human	5`- TGGTAACACGCCCTAACTATC-3`
Scramble shRNA	Human	5`-GATCTCGCTTGGGCGAGAGTAA -3`

Supplementary Figures

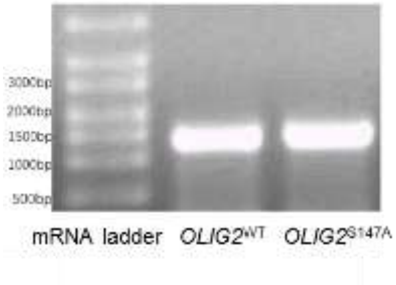

Fig. S1 Agarose gel electrophoresis to detect *OLIG2*^{WT} and *OLIG2*^{S147A}

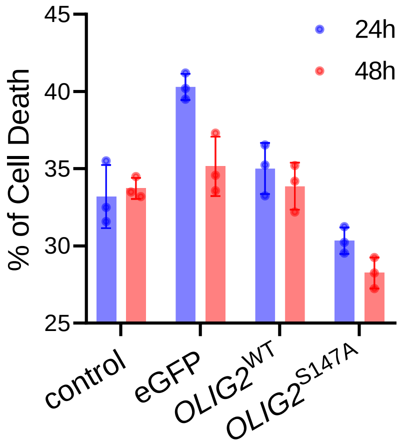

Fig. S2 hiPSC-derived NPCs received one dose (300ng) of eGFP, OLIG2^{WT} or OLIG2*S147A* smRNA transfection or lipid alone as the control. Cells were subjected to CCK8 assay at 24 h and 48h. Data represents mean ± SEM (n = 3).

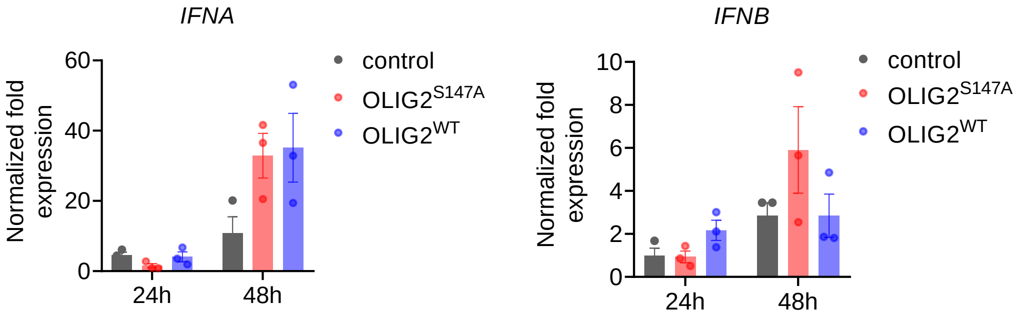

Fig. S3 hiPSC-derived NPCs received 6 daily of *OLIG2*^{WT} or *OLIG2*^{S147A} smRNA transfection or lipid alone as the control. Total RNA was isolated after 24 h and 48 h to measure markers of the IFN signaling (*IFNA*, *IFNB*) by qPCR assay. Data represents mean ± SEM (n = 3).

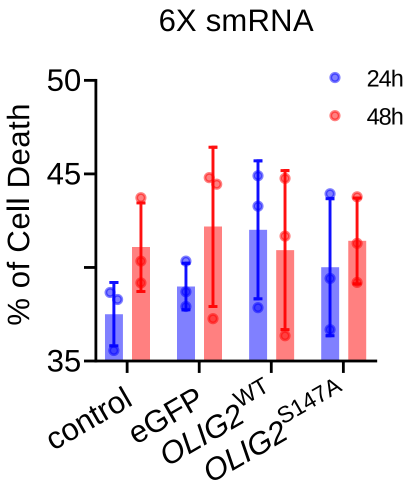

Fig. S4 A hiPSC-derived NPCs received 6 daily (300ng) of *OLIG2*^{WT} or *OLIG2*^{S147A} smRNA transfection or lipid alone as the control. Cells were subjected to CCK8 assay after 24 h and 48h.

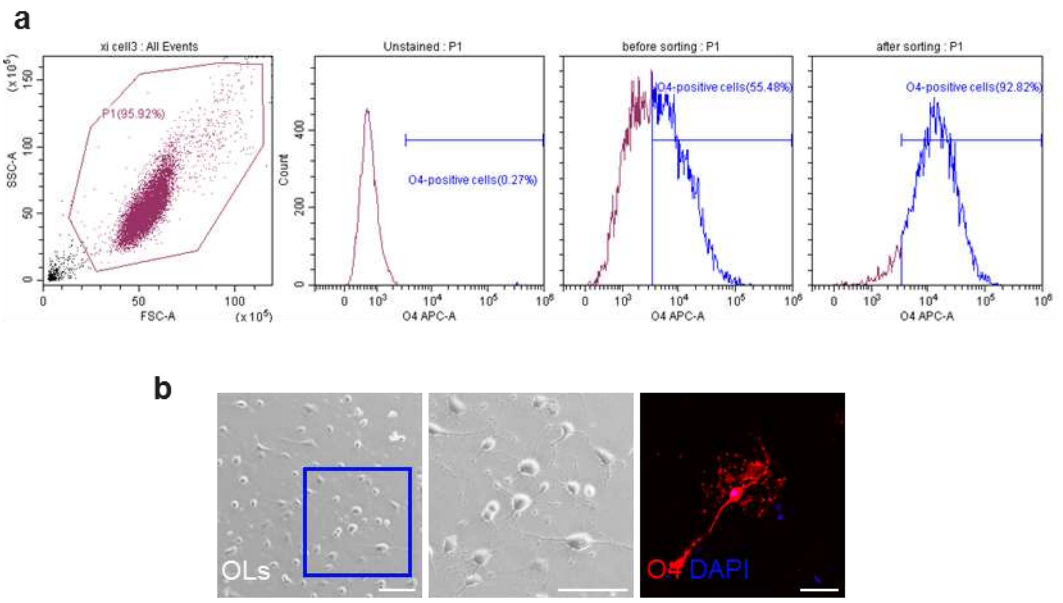

Fig. S5 a Gating strategy for sorting O4^{+} OPCs for cell transplantation. The dot graph

and histogram are representative of 3 independent experiments. **b** After cell sorting, OLIG2S147A smRNA-induced OLs presented with a branched morphology and expressed the more mature oligodendrocyte marker O4, scale bar, 50 μm.

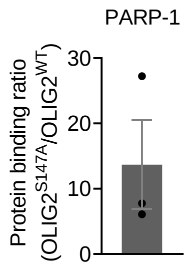

Fig. S6 The *OLIG2*^{S147A}A/*OLIG2*^{WT} binding ratio of PARP-1 (n = 3).

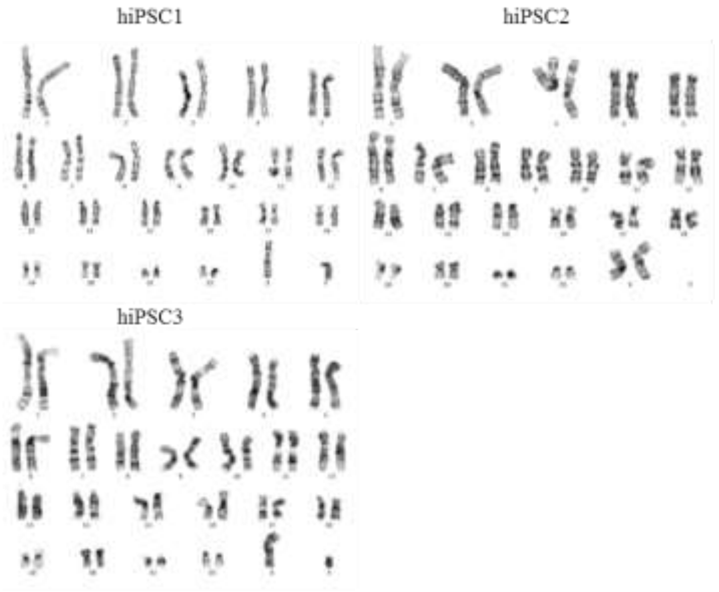

Fig. S7 Cytogenetic analysis of all three hiPSC lines shows a normal karyotype

Uncropped gel blots for Fig.1d

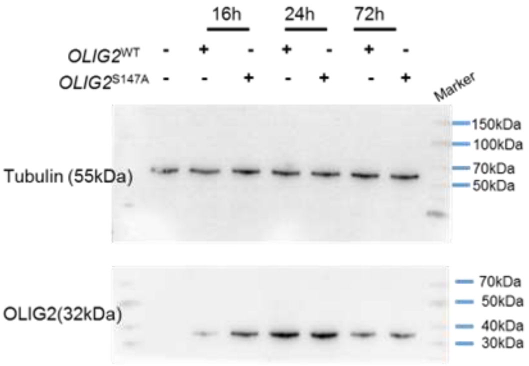

Uncropped gel blots for Fig.5b

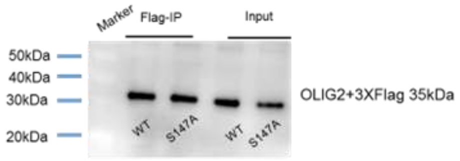

Uncropped gel blots for Fig.6d

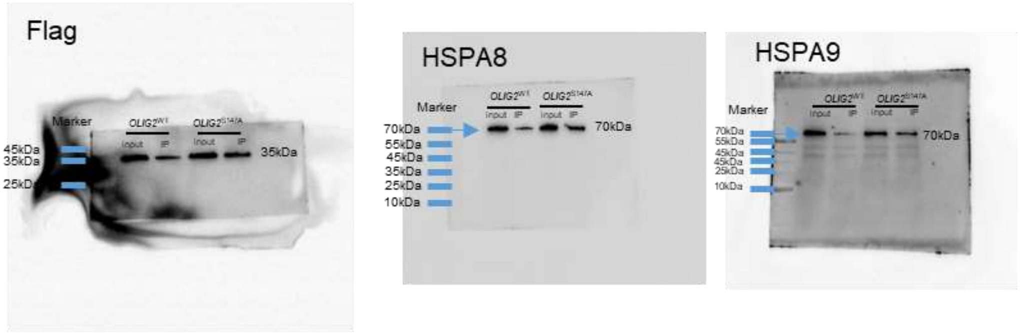

Uncropped gel blots for Fig.6f

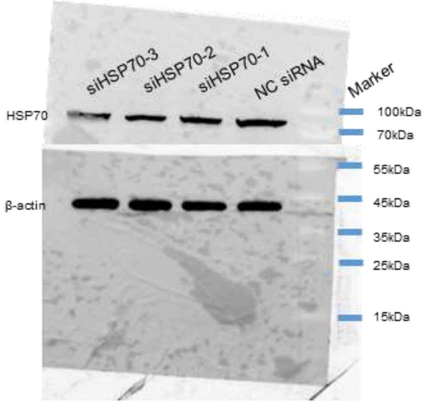

Fig. S8 Original and uncropped gel blots.
