# Supplementary material for: Rapid differentiation of hiPSCs into functional oligodendrocytes using an OLIG2 synthetic modified messenger RNA
Source: Commun Biol. 2022 Oct 14;5:1095. doi: 10.1038/s42003-022-04043-y (PMC9568531; doi:10.1038/s42003-022-04043-y)
Supplement: Supplementary file 2 — Description of Additional Supplementary Files [file 42003_2022_4043_MOESM2_ESM.pdf]

Description of Additional Supplementary Files

File name: Supplementary Data 1

Description: Differential binding proteins

File name: Supplementary Data 2

Description: Pathway enrichment results

File name: Supplementary Data 3

Description: The source data behind the graphs in the paper
